# Supplementary figures and images for: Keystone pathobionts associated with colorectal cancer promote oncogenic reprograming
Source: PLoS One. 2024 Feb 16;19(2):e0297897. doi: 10.1371/journal.pone.0297897 (PMC10871517; doi:10.1371/journal.pone.0297897)

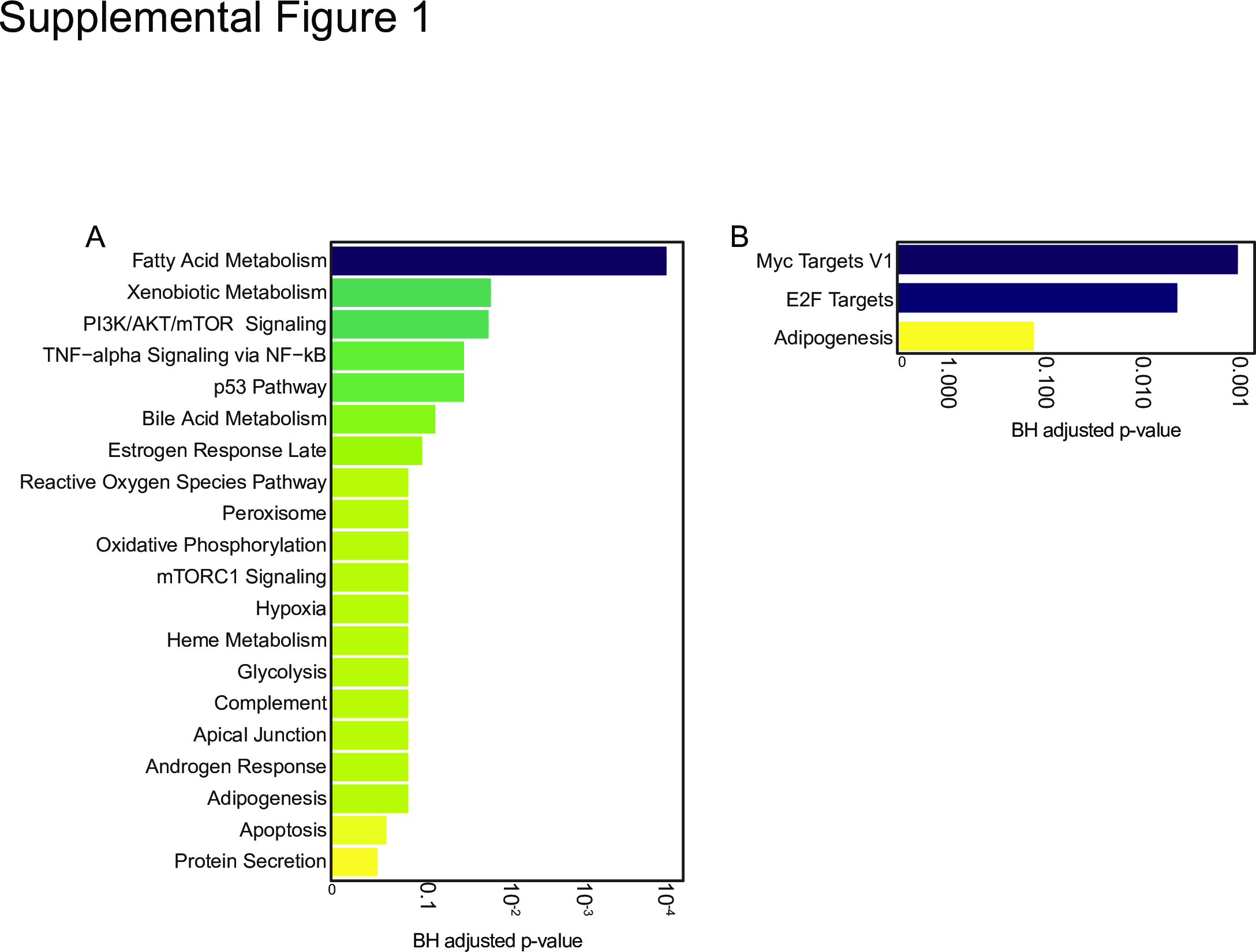

Supplement: S1 Fig — (A) Top 20 differentially enriched pathways (MSigDB Hallmarks 2020) represented in the transcriptomes of cells from CSC-like TA cells from the Fn-exposed ApcMin/+ mouse as compared to the PBS-treated ApcMin/+ mouse. (n = 175 cells, Fisher exact test, BH-FDR-corrected p-values < 0.05, EnrichR) (B) Top 3 differentially enriched pathways (MSigDB Hallmarks 2020) represented in the transcriptomes of cells from CSC-like TA cells from the ETBF-exposed ApcMin/+ mouse as compared to the PBS-treated ApcMin/+ mouse. (n = 175 cells, Fisher exact test, BH-FDR-corrected p-values < 0.05, EnrichR). S1 Fig complements Fig 2. (TIF) [file pone.0297897.s001.tif]

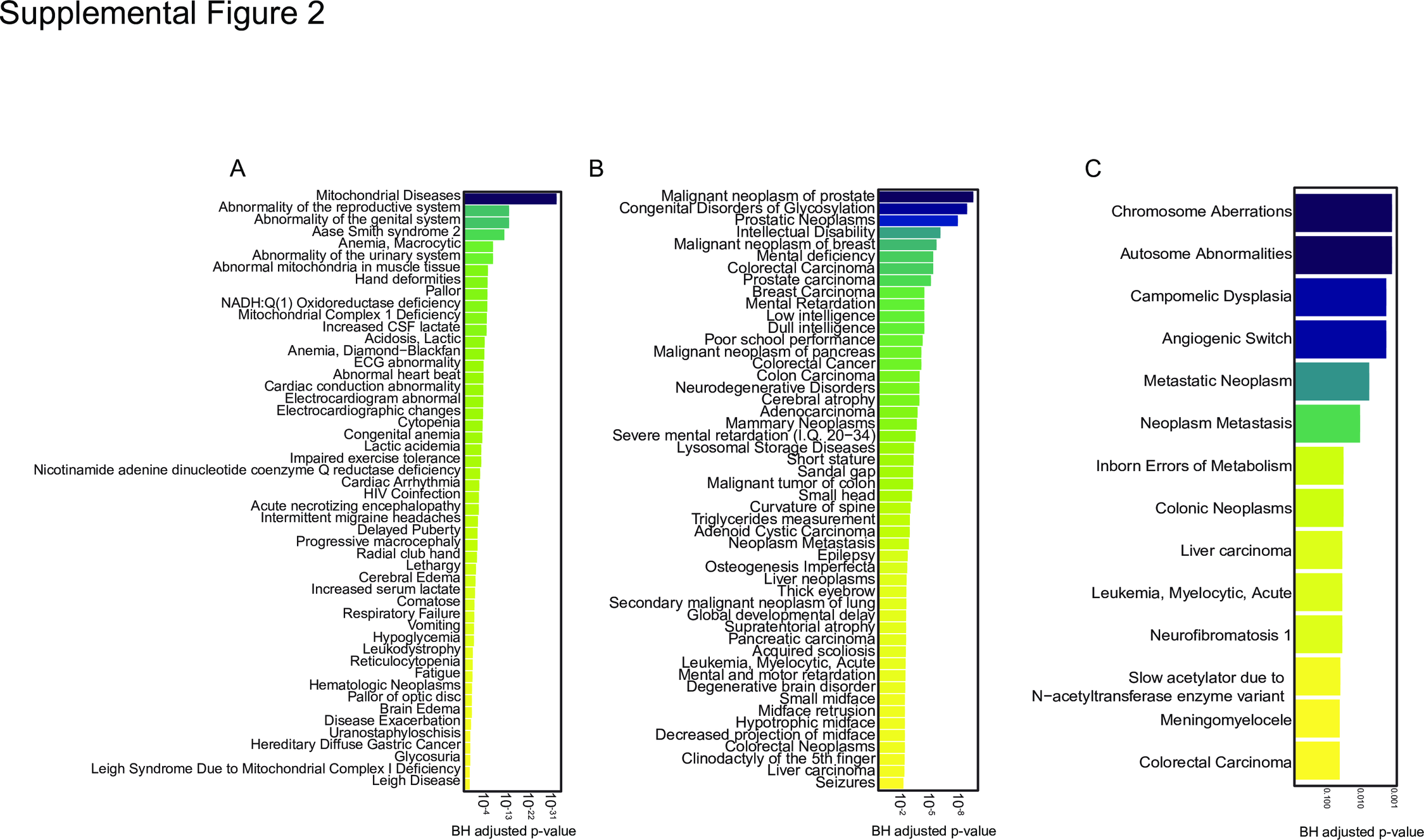

Supplement: S2 Fig — (A) A barplot depicting the top 50 genesets according to DisGeNET (y-axis) for the proliferating TA cells (1), plotted in descending according to corrected p-values (x-axis, Fisher exact test, BH-FDR corrected p-values < 0.05, EnrichR). (B) A barplot depicting the top 50 genesets according to DisGeNET (y-axis) for the proliferating TA cells (2), plotted in descending according to corrected p-values (Fisher exact test, BH-FDR-corrected p-values < 0.05, EnrichR). (C) A barplot depicting the top 14 genesets according to DisGeNET (y-axis) for the late enterocyte progenitors, plotted in descending according to corrected p-values (Fisher exact test, BH-FDR corrected p-values < 0.05, EnrichR). S2 Fig complements Fig 2. (TIF) [file pone.0297897.s002.tif]

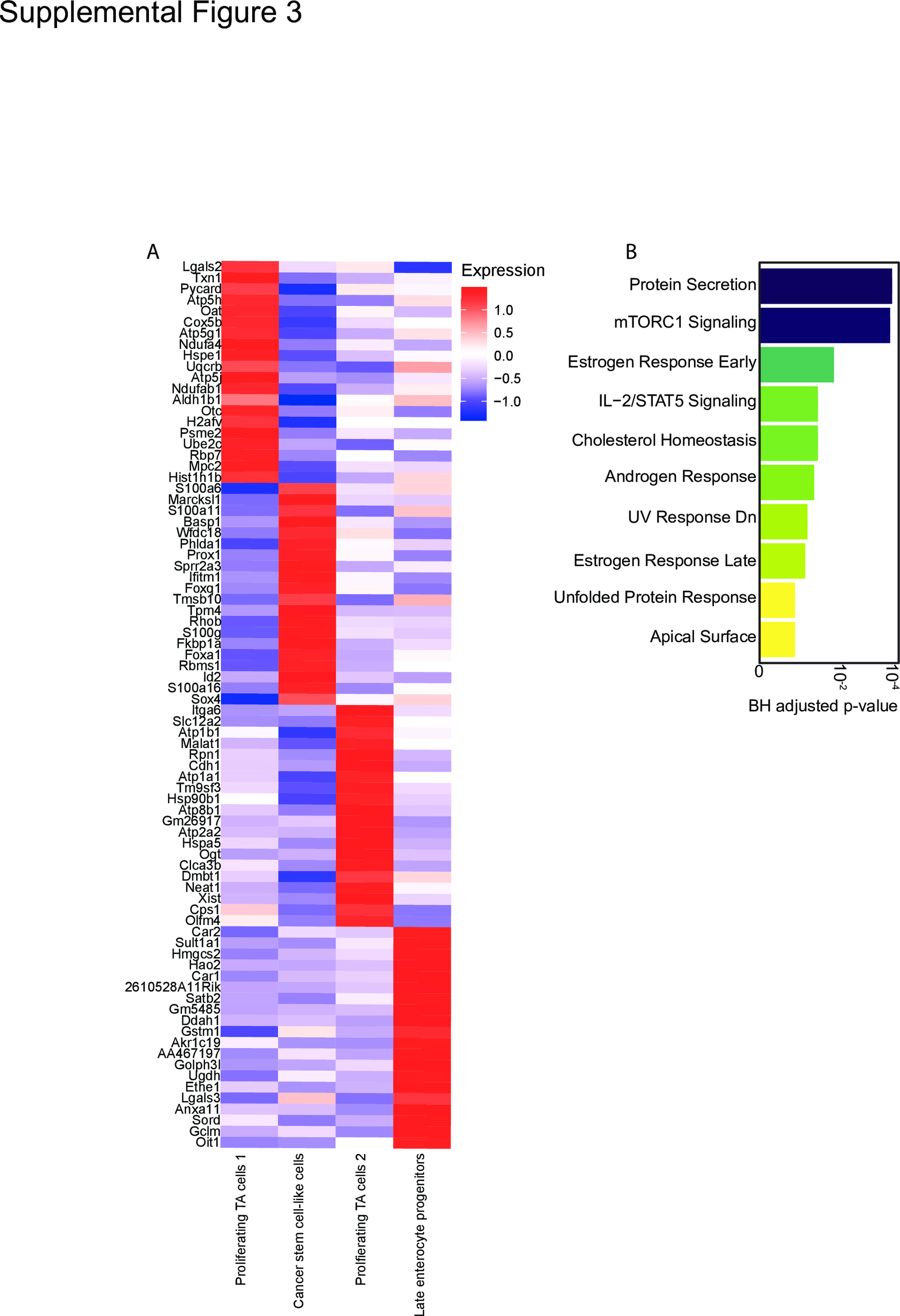

Supplement: S3 Fig — (A) The TA cells depicted here are the 4 subclusters of the complete TA cell population and are an aggregate from all mouse samples (ApcMin/+ mice treated with PBS, Fn or ETBF and wild type mice treated with PBS, Fn or ETBF. A heatmap displaying the top 20 upregulated genes for each TA cluster, log2(fold-change) ≥ 0.25 (Wilcox test), corrected p-value < 0.05 (Bonferroni correction), Seurat), plotted as average expression values (Seurat). (B) Differentially enriched pathways represented in the transcriptomes of proliferating TA cells 2 compared with other TA cell populations. Barplot depicting the top 10 genesets according to the Molecular Signatures Database Hallmark 2020 (MSigDB Hallmarks 2020) for the cancer-like cell population, plotted in descending according to corrected p-values (Fisher exact test, BH-FDR corrected p-values < 0.05, EnrichR). S3 Fig complements Fig 2 and S2 Fig. (TIF) [file pone.0297897.s003.tif]

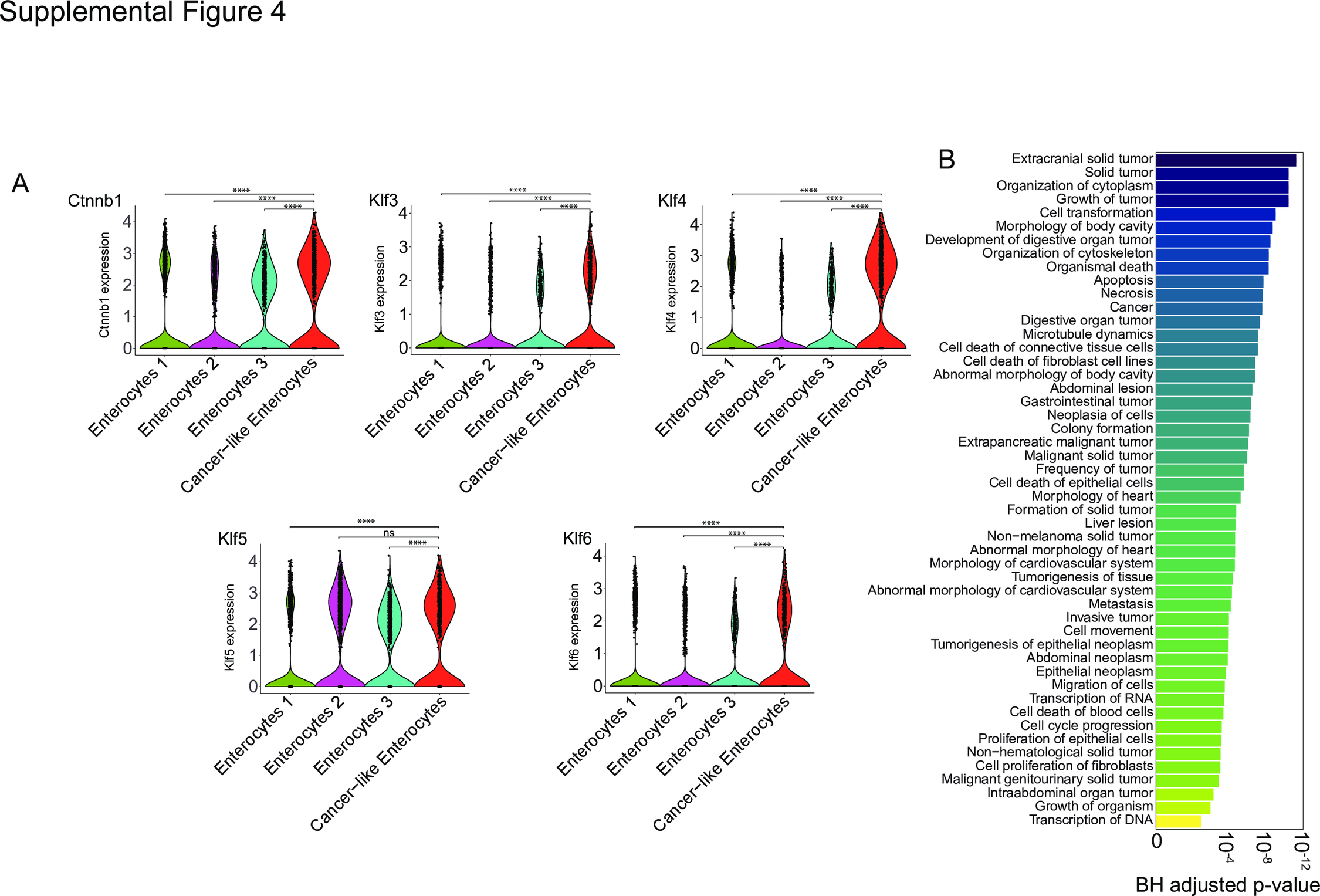

Supplement: S4 Fig — (A) Violin plots displaying selected CRC-associated genes and their expression levels across 4 enterocyte clusters (log2(fold-change) ≥ 0.25, Wilcoxon test, Bonferroni-corrected p-value < 0.05). (B) Barplot depicting the top 50 IPA Diseases and Functions annotations based on corrected p-values (Fisher exact test, BH-FDR corrected p-values < 0.05,) for the cancer-like enterocyte subpopulation. Statistical comparisons were performed using a pairwise Wilcoxon test (* = p ≤ 0.05, ** = p ≤ 0.01, *** = p ≤ 0.001, **** = p ≤ 0.0001), comparing the cancer-like enterocyte population to all other mature enterocyte clusters (see S4 Fig). S4 Fig complements Fig 3. (TIF) [file pone.0297897.s004.tif]

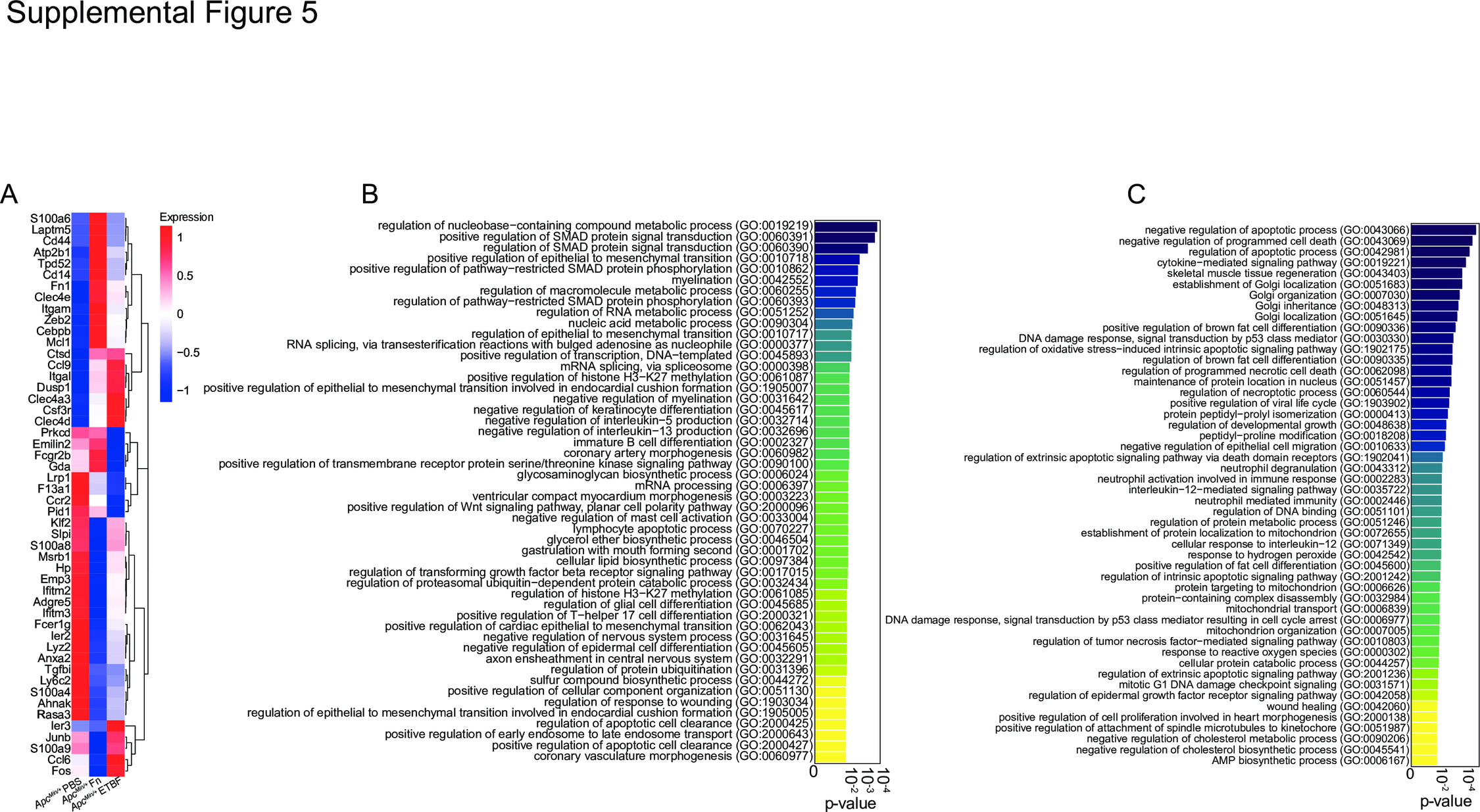

Supplement: S5 Fig — (A) A heatmap displaying the top 50 upregulated genes defining the proinflammatory macrophage population compared across each dataset (log2(fold-change) ≥ 0.25, Wilcoxon Rank Sum test, p-value < 0.05 (unadjusted), Seurat), plotted as average expression values. (B) Barplot depicting the top 50 enriched genesets according to the Gene Ontology Biological Processes 2021 (GOBP21) for proinflammatory macrophages derived from Fn-exposed ApcMin/+ mice when compared to PBS control ApcMin/+ mice, plotted in descending according to p-values (Fisher exact test p-values < 0.05, unadjusted, EnrichR). (C) Barplot depicting the top 50 enriched genesets according to the Gene Ontology Biological Processes 2021 (GOBP21) for proinflammatory macrophages derived from Fn-exposed ApcMin/+ mice when compared to ETBF exposed ApcMin/+ mice, plotted in descending according to p-values (Fisher exact test p-values < 0.05, unadjusted, EnrichR). (TIF) [file pone.0297897.s005.tif]
