# Supplementary material for: Keystone pathobionts associated with colorectal cancer promote oncogenic reprograming
Source: PLoS One. 2024 Feb 16;19(2):e0297897. doi: 10.1371/journal.pone.0297897 (PMC10871517; doi:10.1371/journal.pone.0297897)
Supplement: S1 Table — The top 10 differentially expressed genes per cluster listed were used to classify single-cells into cell types. Marker genes were defined using the FindAllMarkers function in Seurat (log2(fold-change) ≥ 0.25 (Wilcox test), corrected p-value < 0.05 (Bonferroni correction)). The top 10 marker genes were included for each cluster. (DOCX) [file pone.0297897.s006.docx]

**Supplemental Table 1. Genes used to classify single cells.** The top 10 differentially expressed genes per cluster listed were used to classify single-cells into cell types.

| **Cluster** | **Gene** |
| --- | --- |
| B cells | *Igkc, Ighm, Cd74, Iglc2, Mef2c, Cd79a, Ebf1, Ms4a1, H2-Aa, Cd79b* |
| Cholangiocytes | *Defb50, Pbsn, Msmb, Defb1, 9530002B09Rik, Pate9, Dnajc10, Hspa5, Prdx6, Spink1* |
| Enteroendocrine cells | *Sct, Chgb, Chga, Tph1, Neurod1, Cpe, Reg4, Fabp5, Cck, Gip* |
| Epithelial cells | *S100a6, Krt15, Psca, Hspb1, Sprr1a, Thbs1, Anxa1, Fxyd3, Hbegf, Rps21* |
| Erythroid-like and erythroid precursor cells | *Hbb-bs, Hbb-bt, Hba-a2, Hba-a1, Snca, Alas2, Tent5c, Slc25a37, Rsad2, Bpgm* |
| Glandular epithelial cells | *Prol1, Svs5, 2310057J18Rik, Dcpp1, Pate4, Svs4, Mup4, Crisp1, Pip, Clu* |
| Goblet cells | *Zg16, Fcgbp, Muc2, Tﬀ3, Clca1, Spink4, Agr2, Ccl6, Hepacam2, Ido1* |
| Immature Enterocytes 1 | *Slc5a1, Cyp3a11, Mttp, Reg3b, Clca4b, Lct, Anpep, Apoa1, Sis, Apoa4* |
| Mature Enterocytes 1 | *Fabp1, Rbp2, Fabp2, Aldob, Txn1, Gsta1, Aldh1a1, Maoa, Dbi, Gstm3* |
| Mature Enterocytes 2 | *Ly6m, Clca4a, Ada, Slc28a2, Ifrd1, Pmp22, Apoa4, Apob, Krt20, Muc3* |
| Mature Enterocytes 3 | *S100g Sult6b2, Papss2, Fth1, Ncoa4, Nadk2, Ugdh, Slc40a1, Mt1, Mt2* |
| Mature Enterocytes 4 | *Car1, 1810065E05Rik, Saa1, Cyp2c55, Hmgcs2, Car4, Sycn, 2610528A11Rik, Lypd8, Guca2a* |
| Mature Enterocytes 5 | *Cck, Sct, Slc27a4, Dgat1, Ces2a, Anpep, Apoc3, Cox6a1, Apoa4, Apoa1* |
| Memory T cells | *Top2a, Hist1h2ap, Pclaf, Mki67, Stmn1, Tubb5, Tuba1b, Hmgb2, Gzma, Xist* |
| Mesothelial cells | *Dcn, C3, Igfbp6, Rarres2, Slpi, Col3a1, Cavin1, Gas1, Fmo2, Gsn* |
| Myeloid cells | *Lyz2, Cd74, Apoe, H2-Ab1, H2-Eb1, H2-Aa, Apol7c, Aif1, Cst3, Psap* |
| Paneth cells | *Defa30, Gm14851, AY761184, Lyz1, Defa21, Itln1, Defa29, Defa22, Defa24, Clps* |
| Plasmacytoid dendritic cells (pDCs) | *Siglech, Bst2, Irf8, Tcf4, Ly6d, Psap, Mpeg1, Rnase6, Ccr9, Cox6a2* |
| T cells | *Ccl5, Gzma, Gzmb, Cd7, Cd3e, Cd3g, Nkg7, AW112010, Bcl2, Ikzf2* |
| TA cells | *Hmgb2, Stmn1, Ptma, Ube2c, Lgals2, H2afz, Rps2, Ccdc34, Pclaf, Hspd1* |
| Tuft cells | *Cd24a, Hck, Sh2d6, Rgs13, Dclk1, Kctd12, Nrgn, Matk, Eppk1, Krt18* |
